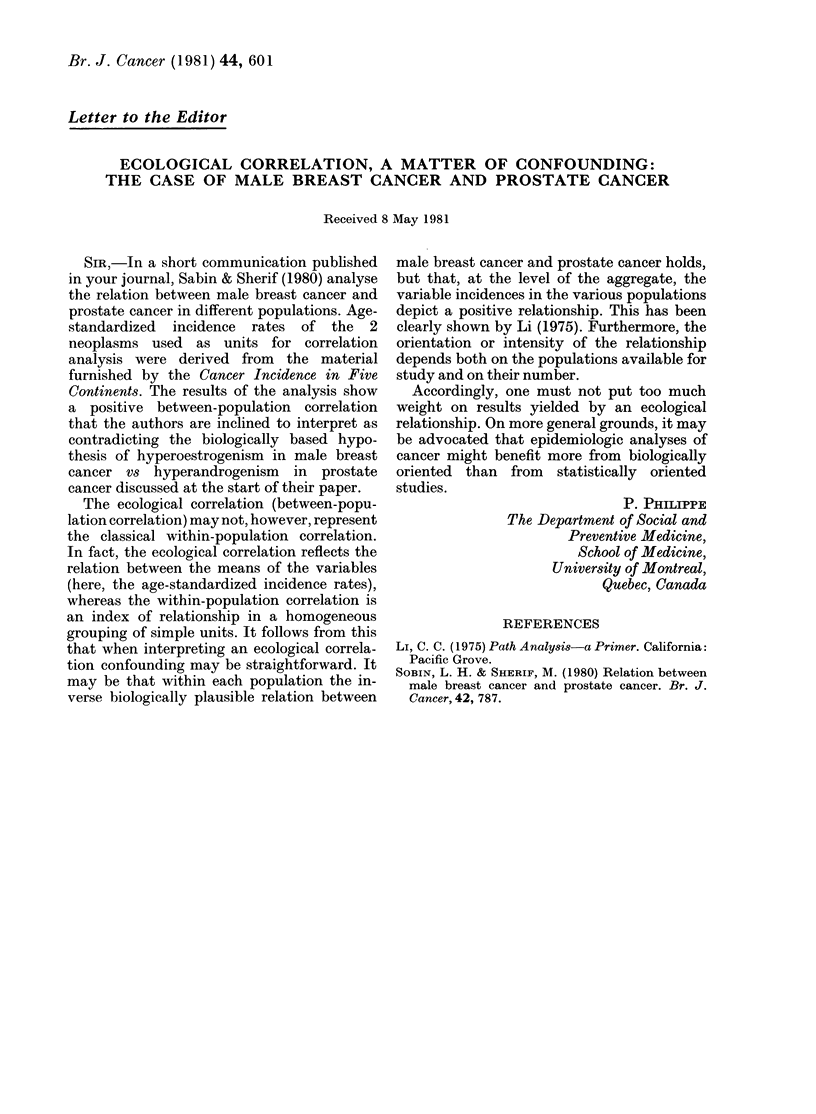# Ecological correlation, a matter of confounding: The case of male breast cancer and prostate cancer

**Published:** 1981-10

**Authors:** P. Philippe


*Br. J. Cancer* (1981) **44**, 601

### *Letter to the Editor*

### ECOLOGICAL CORRELATION, A MATTER OF CONFOUNDING: THE CASE OF MALE BREAST CANCER AND PROSTATE CANCER

Received 8 May 1981

SIR,—In a short communication published in your journal, Sabin & Sherif (1980) analyse the relation between male breast cancer and prostate cancer in different populations. Age-standardized incidence rates of the 2 neoplasms used as units for correlation analysis were derived from the material furnished by the *Cancer Incidence in Five Continents*. The results of the analysis show a positive between-population correlation that the authors are inclined to interpret as contradicting the biologically based hypothesis of hyperoestrogenism in male breast cancer *vs* hyperandrogenism in prostate cancer discussed at the start of their paper.

The ecological correlation (between-population correlation) may not, however, represent the classical within-population correlation. In fact, the ecological correlation reflects the relation between the means of the variables (here, the age-standardized incidence rates), whereas the within-population correlation is an index of relationship in a homogeneous grouping of simple units. It follows from this that when interpreting an ecological correlation confounding may be straightforward. It may be that within each population the inverse biologically plausible relation between male breast cancer and prostate cancer holds, but that, at the level of the aggregate, the variable incidences in the various populations depict a positive relationship. This has been clearly shown by Li (1975). Furthermore, the orientation or intensity of the relationship depends both on the populations available for study and on their number.

Accordingly, one must not put too much weight on results yielded by an ecological relationship. On more general grounds, it may be advocated that epidemiologic analyses of cancer might benefit more from biologically oriented than from statistically oriented studies.

P. PHILIPPE
*The Department of Social and Preventive Medicine,*
*School of Medicine,*
*University of Montreal,*
*Quebec, Canada*

### REFERENCES

LI, C. C. (1975) *Path Analysis—a Primer*. California: Pacific Grove.

SOBIN, L. H. & SHERIF, M. (1980) Relation between male breast cancer and prostate cancer. *Br. J. Cancer*, **42**, 787.